# “Suicide” Proteins Contribute to Sperm Creation

**DOI:** 10.1371/journal.pbio.0020034

**Published:** 2003-12-15

You might say that caspases are obsessed with death. The primary agents of programmed cell death, or apoptosis, caspases kill cells by destroying proteins that sustain cellular processes. Apoptosis, a highly controlled sequence of events that eliminates dangerous or unnecessary cells, contributes to a wide variety of developmental and physiological processes—in a developing embryo, apoptosis creates the space between fingers and adjusts nerve cell populations to match the number of cells they target; in an adult, apoptosis counters cell proliferation to maintain tissue size and density. Now it appears that caspases may also play a role in creating life. As Bruce Hay, Jun Huh, and colleagues report, multiple caspases and caspase regulators are required for the proper formation of free-swimming sperm in the fruitfly Drosophila.

Caspases, which typically exist in a quiescent state in nearly all cells, are regulated through a complex network of activators and inhibitors. Once activated, a “caspase cascade” ultimately cleaves and irreversibly alters the function of essential cellular proteins, leading to apoptosis. A few of the dozen-plus known caspases appear to contribute to inflammation responses, but the vast majority are enlisted to kill cells. Not surprisingly, cells keep caspase activation under tight wraps. That's why it's intriguing that multiple caspases normally associated with the induction of cell death participate in this nonapoptotic process.

During spermatogenesis, germline precursor cells—the cells that generate sex cells—give rise to 64 haploid spermatids. (Sex cells are haploid, containing half the chromosomes found in body cells.) Spermatids are connected by intracellular “bridges” that, along with most other cytoplasmic components, must be expelled in a process called “individualization” to create terminally differentiated free-swimming sperm. Protein structures known as investment cones surround each spermatid nucleus and sweep out the neighboring cytoplasm, bridges, and organelles, forming a bulge that eventually detaches as a “waste bag” as it reaches the sperm tail. This process—elimination of cytoplasm and membrane packaging of individual spermatids—also occurs in mammals. Many types of human infertility result when it is disrupted.

To explore how caspases affect this process, Hay's group studied the consequences of inhibiting caspase activity (or the activity of specific caspase activators) in the male germline cells of fruitflies. In both cases, they observed that the bulges and waste bags were either abnormally small or absent and that the normal path of investment cone movement was disrupted. The researchers also inspected the flies to look for structural differences and found that spermatids in both mutant strains remained connected by cytoplasmic bridges and retained residual cytoplasm. Together, the authors conclude, these results demonstrate that individualization depends on caspase activity.

Hay's team went on to characterize the pathways that activate caspases during sperm individualization. They found that in one pathway, two key activators of caspase-dependent cell death—Ark and Hid (both of which have mammalian counterparts)—promote the activity or stabilization of the caspase Dronc. A second caspase, Dredd, and its activator Fadd (which also have mammalian counterparts) were also found to be important. Double mutants that removed both Dronc and Dredd activity had more severe defects in individualization than mutants that removed only one or the other, suggesting that these caspases have distinct roles in this process. Interestingly, Drice—the downstream caspase activated just as individualization begins (downstream caspases are typically activated by upstream caspases such as Dronc and Dredd)—was not affected by inhibition of Dronc and Dredd. This result, along with the fact that Dronc and Drice were activated at different times and places, suggests that some other mechanism activates Drice. Different apoptosis-related caspases and caspase regulators, the authors conclude, are recruited through different pathways at distinct points in time and space to create individually packaged, free-swimming sperm, a distinctly nonapoptotic process.

Studies in mice suggest that individualization may occur similarly in mammals, with activation of apoptotic caspase cascades resulting in free-swimming sperm and loss of specific caspase activators causing infertility and defective spermatogenesis. The abnormal differentiation and residual cytoplasm seen in caspase-inhibited Drosophila mutants, for example, resemble “cytoplasmic droplet sperm,” a condition seen in infertile men. Insights into the molecular basis of caspase activation in sperm individuation could provide clues to male infertility and suggest possible treatments. Given the widespread role of programmed cell death in supporting processes fundamental to life, perhaps it's not surprising that the agents of apoptosis also support the creation of life.

**Figure:**
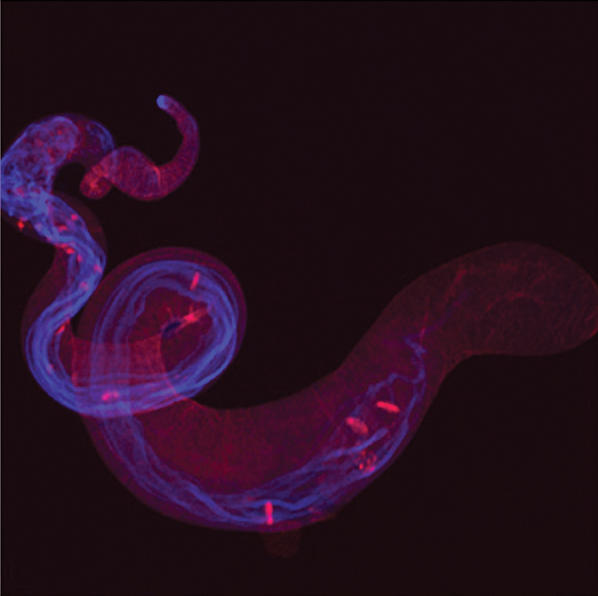
Developing spermatids in a normal Drosophila testis

